# Associations Between PFAS Exposure and HPG Axis Hormones in U.S. Women

**DOI:** 10.3390/life15121923

**Published:** 2025-12-16

**Authors:** Yu-Wei Fang, Ching-Way Chen, Hsuan-Cheng Lin, Ta-Chen Su, Chikang Wang, Chien-Yu Lin

**Affiliations:** 1 Division of Nephrology, Department of Internal Medicine, Shin-Kong Wu Ho-Su Memorial Hospital, Taipei 111, Taiwan; m005916@gmail.com; 2 School of Medicine, College of Medicine, Fu Jen Catholic University, New Taipei 242, Taiwan; 3 Department of Cardiology, National Taiwan University Hospital Yunlin Branch, Yunlin 640, Taiwan; y05213@ms1.ylh.gov.tw; 4 School of Medicine, College of Medicine, Taipei Medical University, Taipei 110, Taiwan; b101114002@tmu.edu.tw; 5 Institute of Environmental and Occupational Health Sciences, College of Public Health, National Taiwan University, Taipei 100, Taiwan; tachensu@ntu.edu.tw; 6 Department of Internal Medicine, National Taiwan University Hospital, Taipei 100, Taiwan; 7 Department of Internal Medicine, College of Medicine, National Taiwan University, Taipei 100, Taiwan; 8 Department of Internal Medicine, Tungs’ Taichung MetroHarbor Hospital, Taichung 435, Taiwan; 9 Department of Environmental Engineering and Health, Yuanpei University of Medical Technology, Hsinchu 300, Taiwan; ckwang@mail.ypu.edu.tw; 10 Department of Internal Medicine, En Chu Kong Hospital, New Taipei 237, Taiwan

**Keywords:** perfluoroalkyl substances, endocrine disruption, gonadotropins, reproductive endocrinology, environmental epidemiology, biomonitoring

## Abstract

**Purpose**: This study aimed to investigate the associations between serum per- and polyfluoroalkyl substances (PFAS) and reproductive hormones, including follicle-stimulating hormone (FSH), anti-Müllerian hormone (AMH), estradiol, and progesterone, in U.S. women. **Approach and Results**: We conducted a cross-sectional study using data from the National Health and Nutrition Examination Survey (NHANES) 2017–2018. The study included 612 women aged ≥18 years with available PFAS and sex hormone measurements. Serum concentrations of four major PFASs (linear perfluorooctanoic acid [n-PFOA], perfluorooctane sulfonic acid [PFOS], perfluorononanoic acid [PFNA], and perfluorohexane sulfonic acid [PFHxS]) were analyzed, along with serum levels of FSH, AMH, estradiol, and progesterone measured by isotope dilution liquid chromatography–tandem mass spectrometry. Higher serum PFAS concentrations were associated with increased FSH and decreased AMH, estradiol, and progesterone. For example, each interquartile range (IQR) increase in ln-PFNA was associated with a 42.0% increase in ln-FSH (*p* = 0.01) and 32.2% lower ln-AMH (*p* < 0.001), 33.0% lower ln-estradiol (*p* = 0.004), and 40.9% lower ln-progesterone (*p* = 0.02). A PFAS exposure index was related to higher FSH and lower AMH, estradiol, and progesterone, with stronger effects in premenopausal women. **Conclusions**: PFAS exposure was linked to broad endocrine disruption in women, with consistent alterations across gonadotropins and sex steroids. These findings suggest that PFAS exposure was associated with hormonal patterns consistent with diminished ovarian reserve and potential changes in reproductive function, underscoring the need for longitudinal studies and regulatory actions to mitigate exposure.

## 1. Introduction

Per- and polyfluoroalkyl substances (PFASs) are widely used synthetic chemicals known for their environmental persistence and bioaccumulation [1]. Human exposure occurs primarily through contaminated drinking water, dietary sources, and consumer products [2]. PFAS are recognized endocrine-disrupting chemicals (EDCs), defined as exogenous substances that interfere with hormone action and endocrine signaling [3]. As EDCs, PFAS may exert biological effects beyond the reproductive system, including disruptions in metabolic regulation, immune function, inflammatory pathways, and developmental processes [4]. These systemic effects are thought to arise through mechanisms such as hormone–receptor interactions, modulation of nuclear receptor signaling, oxidative stress, and immune–endocrine crosstalk [5]. Consistent with these pathways, experimental studies indicate that PFAS can disrupt steroidogenesis, impair ovarian follicle development, and alter hypothalamic–pituitary–gonadal (HPG) axis signaling [6,7,8].

Each reproductive hormone provides distinct mechanistic insight into HPG axis function. FSH reflects pituitary gonadotropin output; estradiol and progesterone represent ovarian steroidogenesis and luteal function [9]; and anti-Müllerian hormone (AMH) serves as a marker of ovarian reserve and granulosa cell activity [10]. Assessing these hormones jointly enables evaluation of potential PFAS-related disruptions across multiple regulatory levels—pituitary signaling, ovarian follicular dynamics, and downstream steroidogenesis—offering substantially greater explanatory power than prior PFAS studies that examined only one or two isolated hormones. Prior epidemiologic studies have evaluated selected reproductive hormones in relation to PFAS exposure, yet the evidence remains fragmented. Earlier NHANES cycles measured only a limited subset of hormones, and analytic approaches varied across survey years due to differences in laboratory assays, hormone panels, and demographic distributions. For example, NHANES 2011–2012 reported no associations between PFAS and testosterone in women [11], whereas analyses from 2013 to 2016 in postmenopausal women found positive associations with total and free testosterone but inverse associations with sex hormone-binding globulin (SHBG) [12]. NHANES 2015–2016 demonstrated positive associations with free testosterone among younger women (20–49 years), while estradiol showed mixed associations among women aged ≥50 years [13]. These inconsistencies likely reflect methodological heterogeneity and the absence of upstream gonadotropins and ovarian reserve markers in earlier cycles. Non-NHANES studies offer complementary but still partial evidence; for instance, PFAS exposure was associated with lower progesterone in a small cohort of reproductive-aged women [14], and with higher FSH and lower estradiol among midlife women in the Study of Women’s Health Across the Nation (SWAN) [15]. However, none of these studies evaluated FSH, AMH, estradiol, and progesterone jointly.

Important gaps remain. NHANES studies have not evaluated upstream gonadotropins such as FSH together with key downstream markers such as AMH, which reflects ovarian reserve [16]. To date, no study has integrated both upstream and downstream reproductive hormones within a unified analytic framework, such as structural equation modeling (SEM), in a nationally representative population of adult women. To address these gaps, we analyzed data from the 2017–2018 NHANES, the only cycle that concurrently measured both PFAS and a comprehensive panel of reproductive hormones. We examined the associations of four major PFASs (linear perfluorooctanoic acid (n-PFOA), perfluorooctane sulfonic acid (PFOS), perfluorononanoic acid (PFNA), and perfluorohexane sulfonic acid (PFHxS)) with key reproductive hormones, including FSH, AMH, estradiol, and progesterone, in women aged 18 years and older. We further applied SEM to assess potential mediating pathways through estradiol, progesterone, and AMH in the relationship between PFAS and FSH. Clarifying these associations is critical, as reproductive hormones not only govern fertility but also influence long-term health outcomes such as cardiovascular disease and osteoporosis in women [17]. This study provides novel insights into how PFAS exposure may influence reproductive hormone regulation in adult women in the general U.S. population.

## 2. Materials and Methods

### 2.1. Study Population

**Figure 1:**
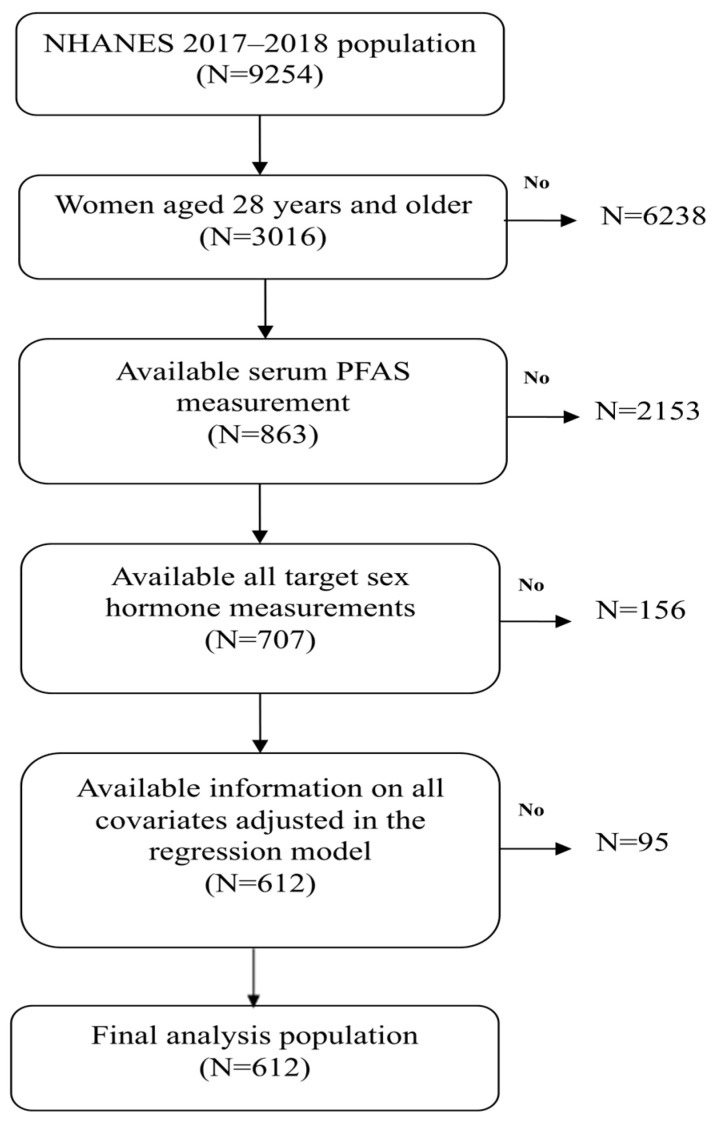
Flowschart algorithm.

NHANES, conducted biennially, is designed to provide a representative assessment of the health and nutritional status of the civilian, non-institutionalized U.S. population. To achieve national representativeness, the survey employs a complex, multistage probability sampling strategy. Detailed descriptions of survey methodology and informed consent procedures are publicly available on the NHANES website [18]. In this study, we used data from the 2017–2018 NHANES, the only cycle that concurrently measured PFAS and the full panel of reproductive hormones. This cycle originally included 9254 participants, of whom 3016 were women aged ≥18 years. Among them, 863 had serum specimens available for PFAS measurements, and 707 had complete information on all targeted sex hormones. After applying covariate eligibility criteria, a final analytic sample of 612 women was retained for multiple regression modeling. The selection process is illustrated in Figure 1.

### 2.2. Measurement of Serum PFAS Levels

In the 2017–2018 NHANES cycle, serum concentrations of nine PFAS were measured in a one-third subsample. For the present analysis, we selected five legacy PFAS (n-PFOA, n-PFOS, sm-PFOS, PFNA, and PFHxS) a priori, based on their high detection frequencies and frequent use in reproductive and endocrine epidemiology. In this study population, detection frequencies, in the same order, were 99.8%, 99.8%, 99.3%, 90.2%, and 99.2%. To facilitate comparability across studies, we combined n-PFOS and sm-PFOS into PFOS for the primary analyses. Branched PFOA isomers were excluded because 92% of values were below the limit of detection (LOD). Laboratory measurements were conducted at the Centers for Disease Control and Prevention (CDC) using automated solid-phase extraction coupled with high-performance liquid chromatography–tandem mass spectrometry (LC–MS/MS). Detailed analytical procedures are documented in the NHANES PFAS Laboratory Procedure Manual [19]. The lower limit of detection (LOD) for all analytes was 0.10 ng/mL, with values below the LOD imputed as LOD divided by the square root of two.

### 2.3. Measurement of Serum Sex Hormone Levels

In the 2017–2018 NHANES cycle, residual serum samples from women aged 18 years and older were analyzed for sex hormones. For the present study, we focused on four hormones: FSH, AMH, estradiol, and progesterone. These analytes were quantified using isotope dilution LC–MS/MS to ensure high analytical accuracy. According to CDC laboratory protocols, the LODs were 0.30 mIU/mL for FSH, 0.03 ng/mL for AMH, 1.72 pg/mL for estradiol, and 0.86 ng/dL for progesterone. Detailed analytical procedures are available on the NHANES website [20].

### 2.4. Covariates

Data on sociodemographic factors such as age, sex, and race/ethnicity were derived from the NHANES database. Body mass index (BMI) was computed by dividing body weight (kg) by the square of height (m^2^). Smoking behavior was categorized into three groups—current smokers, individuals exposed to environmental tobacco smoke (ETS), and non-smokers—based on responses to the standardized NHANES smoking questionnaire [21]. Alcohol consumption was assessed by determining whether participants reported drinking at least 12 alcoholic beverages within the past year. Physical activity was evaluated by linking reported activity types with their corresponding metabolic equivalent of task (MET) scores, in accordance with NHANES protocols, and subsequently stratified into tertiles to reflect low, moderate, and high activity levels [22]. Serum albumin and estimated glomerular filtration rate (eGFR) were additionally considered in sensitivity analyses because of their potential influence on PFAS toxicokinetics and hormone regulation. eGFR was calculated using the Modification of Diet in Renal Disease (MDRD) equation. Menopausal status was defined as post-menopausal if women reported cessation of menses due to “menopause/change of life” for ≥12 months or had both ovaries surgically removed. For women with a prior hysterectomy or missing menstrual data, postmenopause was assigned using biochemical criteria—FSH ≥ 30 mIU/mL and estradiol < 30 pg/mL. [23]. Among postmenopausal women in our analytic sample, 237 were classified using questionnaire-based criteria, and 38 were classified based on biochemical thresholds.

### 2.5. Statistics

All descriptive analyses and regression models were conducted using IBM SPSS Statistics, version 30.0 (IBM Corp., Armonk, NY, USA). We incorporated NHANES sampling weights and accounted for clustering and stratification (primary sampling units and strata) to obtain nationally representative estimates; variances were computed via Taylor series linearization [24]. Because PFAS, hormone, and eGFR concentrations were right-skewed, we analyzed natural log-transformed (ln) values. Survey-weighted geometric mean and 95% confidence interval (CI) were derived by exponentiating the survey-weighted mean and CI from the log scale. Associations between serum PFAS and ln-hormones were estimated using survey-weighted linear regression adjusted for age, race/ethnicity, family poverty income ratio, BMI, smoking, drinking, and physical activity. Because all exposure and outcome variables were ln-transformed, the regression coefficients represent proportional effect sizes commonly used in environmental epidemiology. To enhance the interpretability of effect estimates, all β coefficients from the log-linear models were additionally expressed as percent change per interquartile range (IQR) increase in PFAS exposure. Because PFAS concentrations were modeled on the ln scale, IQR values were derived from the ln-transformed PFAS distributions. Nonlinearity was examined in supplementary models by adding polynomial terms. To evaluate the robustness of our findings, we performed several additional analyses. First, we conducted a sensitivity analysis excluding women whose reproductive hormone levels may be substantially influenced by physiological or exogenous factors. This analysis removed participants who were pregnant or breastfeeding at the time of examination, as well as those who reported using birth control pills or female hormone therapy. Second, to assess the influence of NHANES design features on model stability, we fitted unweighted versions of the primary models using the same covariate set. Comparing weighted and unweighted estimates allowed us to examine the sensitivity of the PFAS–hormone associations to survey weighting and the potential impact of extreme sampling weights. Third, we conducted isomer-specific analyses to determine whether linear and branched PFOS demonstrated differential associations with reproductive hormone levels.

To characterize overall PFAS exposure, we constructed a PFAS exposure index by averaging the z-score-standardized values of the ln-transformed PFAS concentrations (ln–n-PFOA, ln-PFOS, ln-PFNA, ln-PFHxS). This equal-weight index provides a simple summary measure of correlated PFAS and is not intended to imply toxicologic equivalence or the absence of interactions among individual compounds. To assess the robustness of this mixture metric, we additionally performed principal component analysis (PCA) on the same ln-transformed PFAS concentrations. PCA was conducted using the correlation matrix, and component loadings, communalities, and explained variance were examined to characterize the underlying mixture structure. Factor scores for the first principal component (PC1) were generated using the regression method and used as an alternative PFAS mixture measure in sensitivity analyses. We further evaluated potential confounding by extending the primary model to include serum albumin (Model 2), eGFR (Model 3), or both biomarkers (Model 4), given their relevance to PFAS toxicokinetics and their dual roles as potential confounders or intermediates in prior PFAS epidemiologic studies. Effect modification was examined by incorporating interaction terms between the PFAS exposure index and prespecified covariates (age, race/ethnicity, BMI, smoking, drinking). Because reproductive aging substantially influences hormone levels, analyses were also stratified by menopausal status. Finally, adjusted marginal predictions of ln-transformed hormone levels across the observed PFAS exposure index range were obtained using survey-weighted linear regression. Points represent subject-level predicted hormone values, and the solid line represents the model-implied linear trend (with R^2^ estimated from the survey-weighted model).

We conducted an exploratory SEM using PROC CALIS in SAS 9.4 to illustrate the structural relationships among PFAS and reproductive hormones. Because PROC CALIS does not support the full NHANES complex survey design (strata, PSU, and sampling weights simultaneously), the SEM was treated as exploratory. All primary inferences relied on fully survey-weighted regression models, and the SEM was used only to visualize potential pathways among hormones. The PFAS exposure index was specified as the exogenous variable, with FSH, estradiol, progesterone, and AMH treated as endogenous hormone variables within a conceptual path framework. The model included hypothesized association paths linking PFAS exposure to each hormone and among hormones within the HPG axis. The same covariates used in the linear regression models (age, race/ethnicity, family poverty–income ratio, BMI, smoking, drinking, and physical activity) were entered as exogenous predictors of all endogenous hormone variables. Parameters were estimated by generalized least squares in SAS PROC CALIS. Global fit was evaluated using the goodness-of-fit index (GFI), normed fit index (NFI), and root mean square residual (RMR); GFI and NFI ≥ 0.90 together with RMR ≤ 0.05 were interpreted as an acceptable fit. Path coefficients (standardized β), their standard errors, and overall fit statistics are reported. A two-sided *p* < 0.05 was considered statistically significant.

## 3. Results

Table 1 shows the baseline characteristics of the 612 women. The mean age was 49.0 years. The sample included 37.9% non-Hispanic White, 20.3% non-Hispanic Black, and 14.7% Mexican-American individuals; 17.0% were current smokers, and 64.7% reported alcohol consumption ≥ 12 drinks/year. Table 2 demonstrated that serum PFAS concentrations (n-PFOA, PFOS, PFNA, PFHxS) were consistently higher in postmenopausal than in premenopausal women, whereas estradiol, progesterone, and AMH were higher in premenopausal women; FSH was markedly higher postmenopause.

Table 3 shows that higher serum concentrations of individual ln-PFAS were associated with substantial changes in ln-reproductive hormone levels when expressed as percent change per IQR increase in exposure. Specifically, each IQR increase in ln-n-PFOA was associated with a 19.7% increase in ln-FSH (*p* = 0.035), as well as 16.4% lower ln-AMH (*p* = 0.048) and 23.2% lower ln-estradiol (*p* = 0.017). ln-PFOS was also positively associated with ln-FSH, with each IQR increase corresponding to a 31.7% increase (*p* = 0.032), and was inversely associated with ln-estradiol (−29.9%, *p* = 0.002) and ln-progesterone (−47.4%, *p* = 0.003). ln-PFNA showed the strongest associations across the ln-PFAS examined: an IQR increase was linked to a 42.0% higher ln-FSH (*p* = 0.010) and 32.2% lower ln-AMH (*p* < 0.001), 33.0% lower ln-estradiol (*p* = 0.004), and 40.9% lower ln-progesterone (*p* = 0.021). Similarly, ln-PFHxS was positively associated with ln-FSH, with each IQR increase corresponding to a 21.6% increase (*p* = 0.057), and was inversely associated with ln-estradiol (−24.3%, *p* = 0.007) and ln-progesterone (−37.1%, *p* = 0.031). To further explore dose–response patterns, we evaluated potential nonlinear associations, but only the linear terms were statistically significant, indicating that linear models best described these relationships.

Sensitivity analyses excluding pregnancy, breastfeeding, and hormone-use participants yielded results that were consistent with the primary findings (Supplemental Table S1). The direction and magnitude of the associations between PFAS and reproductive hormones remained largely unchanged. For example, each IQR increase in ln-PFNA was associated with higher FSH and lower AMH, estradiol, and progesterone, with effect sizes similar to those observed in the full sample. Estimates for PFOS and n-PFOA also retained their original patterns. Although confidence intervals widened slightly due to the reduced sample size (N = 495), the overall PFAS–hormone associations persisted. To evaluate the influence of NHANES design features, we also compared the primary weighted models with unweighted analyses (Supplemental Table S2). The unweighted models demonstrated effect estimates that were directionally consistent with the weighted results across all PFAS compounds and hormone outcomes. Weighted estimates were generally larger in magnitude, as expected given the correction for sampling probability, but the overall pattern—higher FSH and lower AMH, estradiol, and progesterone with higher PFAS concentrations—remained stable. In addition, isomer-specific analyses of PFOS showed patterns consistent with the main findings (Supplemental Table S3). Each IQR increase in n-PFOS was associated with higher FSH and lower AMH, estradiol, and progesterone, with estimates similar in direction and magnitude to total PFOS. The sm-PFOS demonstrated comparable associations, although with slightly wider confidence intervals. These results indicate that both linear and branched PFOS contribute similarly to the observed PFOS–hormone relationships, supporting the use of combined PFOS in the primary models.

Table 4 demonstrated that a higher PFAS exposure index was associated with notable alterations in ln- serum sex hormone levels when expressed as percent change per IQR increase. Each IQR increase in the PFAS exposure index was associated with a 39.5% increase in ln-FSH (*p* = 0.019) and 22.3% lower ln-AMH (*p* = 0.027), as well as 34.6% lower ln-estradiol (*p* = 0.002) and 49.2% lower ln-progesterone (*p* = 0.014). Significant interactions by age were observed for FSH (*p*_for interaction = 0.038), AMH (*p*_for interaction < 0.001), and progesterone (*p*_for interaction = 0.046), whereas no effect modification was found for ethnicity, BMI, smoking, or drinking. Sensitivity analyses adjusting for serum albumin (Model 2), eGFR (Model 3), or both (Model 4) yielded results that were consistent with the primary model (Supplemental Table S4). The PFAS Exposure Index remained positively associated with FSH and inversely associated with AMH, estradiol, and progesterone across all models. Although effect estimates were modestly attenuated in albumin- and eGFR-adjusted models, the direction and overall magnitude of associations were similar, indicating that adjustment for these biomarkers did not materially change the conclusions. PCA identified a single dominant component (PC1) with high and relatively balanced loadings across all PFAS (0.834–0.884) and communalities ranging from 0.696 to 0.782 (Supplemental Figure S1). PC1 accounted for 75.0% of the total variance, indicating strong shared structure among the four PFAS. When PC1 factor scores were substituted for the PFAS Exposure Index, results remained highly consistent (Supplemental Table S5). The magnitude and direction of PC1-based estimates closely matched those from the PFAS Exposure Index, supporting the robustness of the mixture findings.

**Figure 2:**
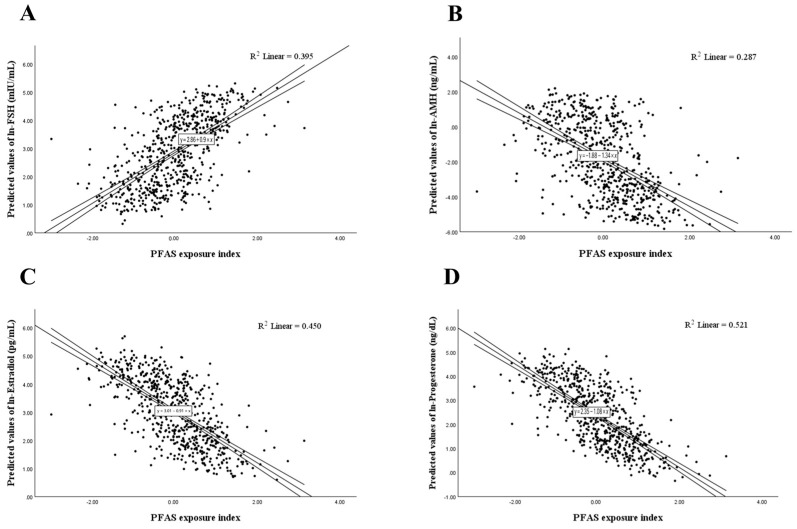
Survey-weighted associations between the PFAS exposure index and predicted ln-sex hormone levels, with fitted regression lines and 95% CI: (**A**) FSH; (**B**) AMH; (**C**) Estradiol; (**D**) Progesterone.

Figure 2 demonstrated dose–response patterns from survey-weighted marginal predictions: the PFAS exposure index was positively related to FSH and negatively related to AMH, estradiol, and progesterone across the observed exposure range.

Table 5 further showed that these associations were mainly evident in premenopausal women. In this group, each IQR increase in the PFAS exposure index was associated with a 54.7% increase in ln-FSH (*p* = 0.010) and 30.7% lower ln-AMH (*p* = 0.004), as well as 38.3% lower ln-estradiol (*p* = 0.002) and 62.7% lower ln-progesterone (*p* = 0.005). In contrast, no significant associations were observed among postmenopausal women.

Supplemental Figure S1 presents the exploratory structural equation model evaluating the associations among the PFAS exposure index, FSH, and reproductive hormones after adjustment for covariates. Across all model specifications, the association path between PFAS exposure and FSH was not statistically significant. In contrast, higher PFAS exposure was associated with lower estradiol and progesterone, and these hormones showed inverse associations with FSH. These patterns were consistent whether estradiol and progesterone were modeled separately or jointly, suggesting that the interrelationships among these hormones within the HPG axis may contribute to the overall association structure. The PFAS–AMH association path was not statistically significant (*p* = 0.116). Model fit indices indicated acceptable fit according to conventional SEM criteria [25], with GFI ranging from 0.89 to 0.91, NFI from 0.90 to 0.92, and RMR from 0.05 to 0.06. Overall, the SEM describes a pattern in which the associations between PFAS exposure and FSH appear to be reflected primarily through their relationships with estradiol and progesterone, rather than a direct statistical association with FSH. These findings reflect the interrelationships among hormones within the HPG axis rather than causal mediation.

## 4. Discussion

In this nationally representative study of U.S. adult women, higher serum concentrations of PFAS were consistently associated with elevated FSH and reduced estradiol, progesterone, and AMH, with associations being more pronounced among premenopausal women. SEM further demonstrated that the relationship between PFAS exposure and FSH was mediated indirectly through reductions in estradiol and progesterone, rather than through direct effects. These findings extend prior epidemiologic research by providing axis-wide evidence of PFAS-related disruption of female reproductive hormones, encompassing both upstream gonadotropins and downstream sex steroids. Notably, this study is the first NHANES-based analysis to include AMH, offering novel insights into how PFAS may compromise ovarian reserve and reproductive function in a nationally representative sample of U.S. women.

Experimental evidence indicates that PFAS exert ovarian toxicity through multiple mechanisms [26]. In human cell line, PFOS/PFOA altered steroidogenic transcripts (including CYP19/aromatase), indicating perturbed estradiol–testosterone balance [27]. In porcine granulosa cells, PFOS and PFOA suppress gonadotropin (Luteinizing Hormone (LH)- and FSH)-stimulated steroidogenesis [28]. In vitro, PFOS and PFOA impair oocyte–granulosa communication and induce oxidative stress, reducing oocyte maturation and survival [29]. Animal studies further demonstrate a reduction in preovulatory follicles, increased follicular atresia, and downregulation of Steroidogenic Acute Regulatory protein (StAR) through decreased histone acetylation, thereby impairing steroidogenesis and reducing estradiol production [30]. Beyond ovarian toxicity, animal studies suggest that PFAS may disrupt the HPG axis. Animal studies suggest that PFOS may impair female reproduction through suppression of ERα-mediated AVPV-kisspeptin signaling and subsequent inhibition of the gonadotropin-releasing hormone–LH surge [8]. Collectively, these data indicate that PFAS can impair female reproduction through multi-level mechanisms, including disrupted oogenesis, altered follicle development, impaired steroidogenesis, and interference with upstream hypothalamic–pituitary signaling.

Previous NHANES 2013–2016 analyses that focused on postmenopausal women reported no associations between PFAS and estradiol, despite observing relationships with total testosterone and SHBG [12]. In our study, the absence of PFAS–estradiol associations after menopause mirrors those results. Notably, we did not evaluate testosterone and SHBG. The difference in hormone panels may partly explain the difference between studies. Another analysis of NHANES 2015–2016 reported that, among adult women, PFAS exposures were positively associated with free testosterone in those aged 20–49 years, whereas among women ≥ 50 years, estradiol showed mixed associations—positive with perfluorodecanoic acid and inverse with PFNA [13]. Because menopausal status was not directly assessed and women were stratified by an age cut point of 50 years, some misclassification is possible. Our study, by applying a specific definition of menopause, helps address this limitation and provides a more refined basis for comparison. In addition, although all of these analyses were based on NHANES, differences in survey cycles, modest sample sizes, and uncontrolled participant characteristics may have contributed to the heterogeneity in effect estimates. Our findings indicate that PFAS exposures were more strongly associated with alterations in sex hormones among women of reproductive age. The absence of PFAS–hormone associations among postmenopausal women is biologically plausible. After menopause, ovarian steroidogenesis is minimal, and hormone levels are low and relatively stable, providing a narrow physiological range in which PFAS-related variation can be detected. In contrast, reproductive-aged women have active folliculogenesis and dynamic HPG-axis feedback, making their sex hormones more susceptible to environmental disruption. Our findings, therefore, align with mechanistic evidence that PFAS interfere with sex-steroid regulation primarily when follicles are hormonally active [26].

Beyond NHANES, longitudinal data from the SWAN cohort of midlife women (ages 45–56, during the menopausal transition) reported positive associations between PFAS and FSH and inverse associations between PFAS and estradiol [15]. These results are consistent with ours. Extending this evidence, our SEM suggests that lower estradiol partially mediates the association between PFAS exposure and higher FSH. Although prior animal studies have indicated that PFAS may also affect upstream hormonal regulation [8], this triangulation across study designs strengthens the biological plausibility that PFAS perturb ovarian steroidogenesis and trigger compensatory pituitary up-regulation of FSH. Nonetheless, our SEM is based on cross-sectional data, and causal direction cannot be established; repeated hormone measurements and further longitudinal mediation are needed.

In this study, we also observed that higher PFAS exposure was associated with decreased AMH and progesterone. To our knowledge, this is the first epidemiologic investigation to evaluate the relationships of PFAS with this hormone. AMH, secreted by granulosa cells of pre-antral and small antral follicles, reflects ovarian reserve [16]. PFAS exposure has been shown to dysregulate AMH signaling by altering its transcription and epigenetic regulation and inducing granulosa cell apoptosis and reduced follicular reserve [31]. In SEM, the direct association between PFAS and AMH was attenuated, while AMH retained an inverse link with FSH. The strong inverse relationship between AMH and FSH, combined with the modest sample size, may have limited our ability to detect a direct PFAS-AMH pathway. Taken together, the inclusion of AMH in our analysis provides the first NHANES-based evidence linking PFAS exposure to ovarian reserve, thereby extending prior NHANES and cohort studies that lacked this critical follicular biomarker. Progesterone, primarily produced by the corpus luteum, plays a critical role in ovulation and luteal function [16]. Experimental studies have shown that PFAS mixtures can enhance FSH-stimulated progesterone secretion and upregulate StAR protein expression in human primary granulosa cells [32]. A study of 178 healthy women aged 25–35 years further reported that PFOS and its salt were inversely associated with salivary progesterone among nulliparous women [14]. In our analysis, progesterone displayed patterns similar to estradiol in SEM, suggesting it may serve as a sensitive indicator of PFAS-related disruption in ovarian steroidogenesis and complement the FSH response. The inclusion of AMH and progesterone provides novel insights into how PFAS could influence and compromise both ovarian reserve and luteal function, highlighting multiple pathways through which these environmental contaminants may disrupt female reproductive endocrinology.

Our findings regarding PFAS are consistent with a broader body of evidence showing that other endocrine-disrupting chemicals also interfere with female reproductive function. For example, bisphenols—another widely studied class of environmental contaminants—have been linked to altered ovarian steroidogenesis, impaired folliculogenesis, and disrupted HPG-axis signaling [33]. Recent reviews highlight that bisphenol exposure may impair fertility through mechanisms such as oxidative stress, epigenetic modification, and interference with hormone receptor pathways [34]. These parallels suggest that PFAS may be part of a wider group of environmental toxicants capable of perturbing reproductive endocrine regulation. The patterns identified in this study hold relevance for both clinical care and public health. The observed associations of PFAS with elevated FSH and reduced estradiol and progesterone may reflect hormonal patterns consistent with diminished ovarian reserve and potential changes in reproductive function, although these cross-sectional data cannot determine causality or directionality. Hormonal declines in estradiol and progesterone not only impair fertility but also contribute to menopausal transition and increased risks of cardiovascular disease, osteoporosis, and metabolic disorders [17]. From a public health perspective, these results underscore the importance of continued biomonitoring of PFAS, regulatory efforts to reduce exposures, and consideration of endocrine endpoints in risk assessment frameworks. Clinically, heightened awareness of environmental exposures as potential modifiers of reproductive hormone profiles may inform counseling and management strategies for women at risk of infertility or early menopause.

This study benefits from the use of NHANES data, which are collected through a nationally representative sampling framework and uniform laboratory protocols, thereby enhancing the validity of findings and their applicability to U.S. adult women. Multiple reproductive hormones were assessed simultaneously, including both upstream gonadotropins (FSH) and downstream sex steroids (estradiol and progesterone) as well as AMH, offering an axis-wide perspective on ovarian function. Furthermore, the application of SEM provided a novel analytic approach to disentangle direct and indirect pathways of endocrine disruption.

Several limitations should also be noted. First, the cross-sectional design prevents causal inference, and hormone levels were measured at a single time point, which may not reflect intra-individual variability across the menstrual cycle or over time. In particular, estradiol and progesterone vary substantially by menstrual cycle phase, and NHANES does not provide complete or reliable information on cycle timing. AMH is also strongly age-dependent, and its steep age-related decline may contribute to residual variability even after statistical adjustment. Second, although major covariates were adjusted for and sensitivity analyses excluded pregnancy, lactation, and hormone use, residual confounding may remain because we did not additionally control for other medications that can influence reproductive hormone levels, including fertility agents, glucocorticoids, thyroid medications, antiepileptic drugs, and other endocrine-active therapies. Third, we also acknowledge that the cross-sectional NHANES design precludes establishing temporality, and that reverse causation or immune-mediated pathways—such as PFAS-related modulation of inflammatory signaling that may secondarily affect ovarian function [5]—cannot be ruled out. Fourth, we did not apply false discovery rate correction given the hypothesis-generating nature of this study, and stringent adjustments may have obscured potentially meaningful associations among hormones within the same regulatory axis. Fifth, our analysis focused on four core hormones along the HPG axis, consistent with our a priori objective of evaluating. Although SHBG and CRP are available in NHANES 2017–2018, including them would broaden the scope beyond HPG-axis regulation and substantially increase the number of comparisons. Other informative biomarkers—such as androgens, thyroid hormones, and inhibin B—were not measured in this NHANES 2017–2018 cycle and therefore could not be evaluated. Finally, SEM could not incorporate the full NHANES design because current SEM software does not support simultaneous use of primary sampling units, strata, and sampling weights. The SEM results should, therefore, be interpreted as exploratory.

## 5. Conclusions

In this nationally representative study of U.S. adult women, higher PFAS exposure was consistently associated with increased FSH and reduced estradiol, progesterone, and AMH, with stronger associations observed in premenopausal women. SEM demonstrated that the PFAS–FSH association was largely mediated by estradiol and progesterone, highlighting indirect endocrine-disrupting pathways along the HPG axis. These findings extend prior NHANES and cohort-based analyses by providing the first NHANES-based evaluation incorporating both upstream gonadotropins and downstream ovarian hormones, including AMH, in adult women. Our results indicate that PFAS exposure was associated with hormonal patterns consistent with diminished ovarian reserve and altered reproductive function. These associations should be interpreted cautiously, and longitudinal studies are needed to clarify temporal relationships.

## Figures and Tables

**Table 1:** Baseline characteristics of study participants (N = 612).

Variables	Mean/Numbers	SD/%
Age (year)	49.01	15.75
Ethnicity		
Mexican-American	90	14.7
Other Hispanic	55	9.0
Non-Hispanic white	232	37.9
Non-Hispanic black	124	20.3
Non-Hispanic Asian	79	12.9
Other ethnicities	32	5.2
Smoking status		
Current smoker	104	17.0
Environmental tobacco smoke	102	16.7
Non-smoker	406	66.3
Alcohol consumption ≥ 12 drinks/year	396	64.7
BMI (kg/m^2^)	30.88	8.86
Physical activity (MET-hours/day)	10.26	16.95

Abbreviations: MET, Metabolic Equivalent of Task.

**Table 2:** Survey-weighted geometric mean and 95% CI of PFAS and sex hormones in the study population, stratified by menopausal status.

	Total (N = 612)	Premenopause (N = 337)	Postmenopause (N = 275)
	Geometric Mean (95% CI)	Geometric Mean (95% CI)	Geometric Mean (95% CI)
PFAS (ng/mL)			
*n*-PFOA	1.20 (1.07–1.33)	1.01 (0.87–1.18)	1.50 (1.35–1.66)
PFOS	3.53 (3.18–3.93)	2.68 (2.40–2.98)	5.11 (4.36–5.98)
PFNA	0.40 (0.33–0.48)	0.32 (0.25–0.41)	0.54 (0.45–0.65)
PFHxS	0.82 (0.75–0.91)	0.63 (0.53–0.74)	1.18 (1.09–1.29)
Sex hormones			
FSH (mIU/mL)	16.27 (13.54–19.54)	5.98 (4.86–7.35)	61.03 (55.58–67.01)
AMH (ng/mL)	0.17 (0.12–0.24)	0.81 (0.53–1.24)	0.02 (0.02–0.02)
Estradiol (pg/mL)	21.48 (17.83–25.88)	56.76 (47.10–68.40)	5.95 (5.12–6.92)
Progesterone (ng/dL)	11.97 (9.30–15.39)	33.69 (25.48–44.55)	3.00 (2.40–3.75)

Weighted population estimates: Total = 94,746,592; Premenopause = 53,921,302; Postmenopause = 40,825,290. Abbreviations: AMH: anti-Müllerian hormone; FSH: follicle-stimulating hormone; n-PFOA: linear perfluorooctanoic acid; PFHxS: perfluorohexane sulfonic acid; PFNA: perfluorononanoic acid; PFOS: perfluorooctane sulfonic acid.

**Table 3:** Percent change in ln-serum sex hormone levels per IQR increase (95% CI) in ln-serum PFAS concentrations in women aged ≥18 years, with results weighted for the sampling strategy.

	FSH (mIU/mL)		AMH (ng/mL)		Estradiol (pg/mL)		Progesterone (ng/dL)	
PFAS (ng/mL)	% Change per IQR (95% CI)	*p* Value	% Change per IQR (95% CI)	*p* Value	% Change per IQR (95% CI)	*p* Value	% Change per IQR (95% CI)	*p* Value
n-PFOA	19.7 (3.2, 38.8)	0.035	−16.4 (−29.3, −1.3)	0.048	−23.2 (−37.4, −5.9)	0.017	−35.2 (−58.5, 1.0)	0.068
PFOS	31.7 (5.2, 64.9)	0.032	−12.9 (−30.4, 9.1)	0.236	−29.9 (−41.5, −16.1)	0.002	−47.4 (−63.3, −24.6)	0.003
PFNA	42.0 (11.1, 81.5)	0.010	−32.2 (−41.5, −21.4)	<0.001	−33.0 (−46.3, −16.5)	0.004	−40.9 (−60.1, −12.5)	0.021
PFHxS	21.6 (1.4, 45.8)	0.057	−4.0 (−18.3, 12.8)	0.592	−24.3 (−36.8, −9.2)	0.007	−37.1 (−57.1, −7.7)	0.031

Model adjusted for age, ethnicity, family poverty income ratio, BMI, smoking status, drinking status, and physical activity. Abbreviations: AMH: anti-Müllerian hormone; FSH: follicle-stimulating hormone; n-PFOA: linear perfluorooctanoic acid; PFHxS: perfluorohexane sulfonic acid; PFNA: perfluorononanoic acid; PFOS: perfluorooctane sulfonic acid.

**Table 4:** Survey-weighted percent change in ln-serum sex hormones per IQR increase (95% CI) in the PFAS exposure index, with interaction *p*-values (age, ethnicity, BMI, smoking, drinking).

	PFAS Exposure Index *						
	% Change per IQR (95% CI)	*p* Value	*p* for Interaction				
			Age	Ethnicity	BMI	Smoking	Drinking
FSH (mIU/mL)	39.5 (8.9, 78.7)	0.019	0.038	0.126	0.551	0.638	0.925
AMH (ng/mL)	−22.3 (−36.6, −4.9)	0.027	<0.001	0.881	0.295	0.285	0.723
Estradiol (pg/mL)	−34.6 (−47.8, −18.1)	0.002	0.090	0.911	0.196	0.954	0.730
Progesterone (ng/dL)	−49.2 (−68.3, −18.5)	0.014	0.046	0.821	0.315	0.750	0.343

Model adjusted for age, ethnicity, family poverty income ratio, BMI, smoking status, drinking status, and physical activity. Abbreviations: AMH: anti-Mullerian hormone; FSH: follicle-stimulating hormone. * PFAS exposure index: the average of standardized (z-score transformed) concentrations of *n*-PFOA, total-PFOS, PFNA, and PFHxS.

**Table 5:** Percent change in ln-serum sex hormones per IQR increase in the PFAS exposure index in individuals aged 18 years and older in women, stratified by menopausal status, with results weighted for the sampling strategy.

	PFAS Exposure Index *			
	Premenopause (N = 337)		Postmenopause (N = 275)	
	% Change per IQR (95% CI)	*p* Value	% Change per IQR (95% CI)	*p* Value
FSH (mIU/mL)	54.7 (15.5, 107.2)	0.010	3.5 (−11.6, 21.2)	0.681
AMH (ng/mL)	−30.7 (−43.4, −15.2)	0.004	3.5 (−3.3, 10.7)	0.397
Estradiol (pg/mL)	−38.3 (−52.9, −19.1)	0.002	−10.8 (−27.2, 9.2)	0.315
Progesterone (ng/dL)	−62.7 (−79.2, −33.1)	0.005	−5.6 (−22.9, 15.6)	0.606

Model adjusted for age, sex, ethnicity, family poverty income ratio, BMI, smoking status, drinking status, and physical activity. Abbreviations: FSH: follicle-stimulating hormone. * PFAS exposure index: the average of standardized (z-score transformed) concentrations of *n*-PFOA, total-PFOS, PFNA, and PFHxS.

## Data Availability

The datasets analyzed in this work are publicly accessible via the NHANES database, maintained by the U.S. Centers for Disease Control and Prevention: https://wwwn.cdc.gov/nchs/nhanes/default.aspx (accessed on 30 September 2025).

## Supplementary Materials

The following supporting information can be downloaded at https://www.mdpi.com/article/10.3390/life15121923/s1. Table S1: Percent change in ln-serum sex hormone levels per IQR increase in ln-serum PFAS concentrations (95% CI) among women aged ≥18 years after excluding participants who were pregnant or breastfeeding at the time of examination, ever using birth control pills or female hormone therapy, with results weighted for the NHANES sampling design (N = 495). Table S2: Percent change in ln-serum sex hormone levels per IQR increase in ln-serum PFAS concentrations (95% CI) among women aged ≥18 years, with results unweighted (N = 612). Table S3: Percent change in ln-serum sex hormone levels per IQR increase in ln-serum PFOS isomers concentrations (95% CI) among women aged ≥18 years with results weighted for the NHANES sampling design (N = 612). Table S4: Percent change in ln-serum sex hormone levels per IQR increase in PFAS Exposure Index concentrations (95% CI) among women aged ≥18 years in different models, with results weighted for the NHANES sampling design. Table S5: Percent change in ln-serum sex hormone levels per IQR increase in the PFAS Exposure Index and principal component 1 (95% CI) among women aged ≥18 years across alternative models, with results weighted for the NHANES sampling design. Figure S1: Exploratory SEM illustrating the associations among the PFAS exposure index (exogenous variable), FSH (mIU/mL), estradiol (pg/mL), progesterone (ng/mL), and AMH (ng/mL). Paths represent hypothesized association structures after covariate adjustment. Panels display models including: (A) estradiol; (B) progesterone; (C) AMH; (D) estradiol and progesterone.

## Abbreviations

AMH	Anti-Müllerian hormone
BMI	Body mass index
CDC	Centers for Disease Control and Prevention
eGFR	Estimated glomerular filtration rate
ETS	Environmental tobacco smoke
FSH	Follicle-stimulating hormone
GFI	Goodness-of-fit index
HPG	Hypothalamic–pituitary–gonadal
IQR	Interquartile range
LC–MS/MS	Liquid chromatography–tandem mass spectrometry
LH	Luteinizing hormone
Ln	Natural log transformed
LOD	Limit of detection
NFI	Normed fit index
NHANES	National Health and Nutrition Examination Survey
PCA	Principal component analysis
PFAS	Per- and polyfluoroalkyl substances
PFHxS	Perfluorohexane sulfonic acid
PFNA	Perfluorononanoic acid
PFOS	Perfluorooctane sulfonic acid
n-PFOA	Linear perfluorooctanoic acid
RMR	Root mean square residual
SEM	Structural equation modeling
SHBG	Sex hormone-binding globulin
StAR	Steroidogenic acute regulatory protein
SWAN	Study of Women’s Health Across the Nation
