# Supplementary material for: Associations Between PFAS Exposure and HPG Axis Hormones in U.S. Women
Source: Life (Basel). 2025 Dec 16;15(12):1923. doi: 10.3390/life15121923 (PMC12734433; doi:10.3390/life15121923)

Supplementary Material

Supplemental Table S1. Percent change in ln-serum sex hormone levels per IQR increase in ln-serum PFAS concentrations (95% CI) among women aged ≥18 years after excluding participants who were pregnant or breastfeeding at the time of examination, ever using birth control pills or female hormone therapy, with results weighted for the NHANES sampling design (N=495).

	**FSH (mIU/mL)**		**AMH (ng/mL)**		**Estradiol (pg/mL)**		**Progesterone (ng/dL)**	
**PFAS (ng/mL)**	**% Change per IQR (95% CI)**	***p* Value**	**% Change per IQR (95% CI)**	***p* Value**	**% Change per IQR (95% CI)**	***p* Value**	**% Change per IQR (95% CI)**	***p* Value**
n-PFOA	18.8 (1.4, 39.2)	0.049	-13.0 (-26.1, 2.5)	0.116	-17.5 (-33.75, 2.7)	0.106	-34.0 (-59.2, 6.6)	0.110
PFOS	36.9 (5.3, 77.9)	0.032	-16.1 (-32.8, 4.8)	0.140	-33.8 (-45.79, -19.2)	0.001	-50.8 (-67.6, -25.3)	0.005
PFNA	42.4 (10.0, 84.2)	0.017	-31.2 (-41.2, -19.4)	<0.001	-32.4 (-47.21, -13.3)	0.008	-42.6 (-64.9, -6.2)	0.043
PFHxS	27.5 (2.6, 58.5)	0.046	-5.8 (-19.3, 10.0)	0.465	-27.04 (-40.3, -10.9)	0.008	-43.5 (-63.3, -13.1)	0.020

Model adjusted for age, ethnicity, family poverty income ratio, BMI, smoking status, drinking status, physical activity. Abbreviations: AMH: anti-Müllerian hormone; FSH: follicle-stimulating hormone; n-PFOA: linear perfluorooctanoic acid; PFHxS: perfluorohexane sulfonic acid; PFNA: perfluorononanoic acid; PFOS: perfluorooctane sulfonic acid.

Supplemental Table S2. Percent change in ln-serum sex hormone levels per IQR increase in ln-serum PFAS concentrations (95% CI) among women aged ≥18 years, with results unweighted (N=612).

	**FSH (mIU/mL)**		**AMH (ng/mL)**		**Estradiol (pg/mL)**		**Progesterone (ng/dL)**	
**PFAS (ng/mL)**	**% Change per IQR (95% CI)**	***p* Value**	**% Change per IQR (95% CI)**	***p* Value**	**% Change per IQR (95% CI)**	***p* Value**	**% Change per IQR (95% CI)**	***p* Value**
n-PFOA	2.2 (-7.8, 13.5)	0.676	-5.3 (-17.1, 8.2)	0.424	-19.1 (-30.2, -6.6)	0.004	-22.1 (-37.0, -3.9)	0.020
PFOS	8.1 (-3.2, 20.7)	0.167	-8.1 (-20.3, 5.8)	0.237	-18.6 (-30.1, -5.1)	0.009	-28.9 (-43.0, -11.3)	0.003
PFNA	13.4 (1.5, 26.6)	0.027	-21.5 (-31.9, -9.5)	<0.001	-23.6 (-34.6, -10.8)	<0.001	-23.9 (-39.3, -4.6)	0.017
PFHxS	5.5 (-4.4, 16.5)	0.292	-0.3 (-12.2, 13.2)	0.960	-17.6 (-28.2, -5.5)	0.006	-23.4 (-37.3, -6.5)	0.009

Model adjusted for age, ethnicity, family poverty income ratio, BMI, smoking status, drinking status, physical activity. Abbreviations: AMH: anti-Müllerian hormone; FSH: follicle-stimulating hormone; n-PFOA: linear perfluorooctanoic acid; PFHxS: perfluorohexane sulfonic acid; PFNA: perfluorononanoic acid; PFOS: perfluorooctane sulfonic acid.

Supplemental Table S3. Percent change in ln-serum sex hormone levels per IQR increase in ln-serum PFOS isomers concentrations (95% CI) among women aged ≥18 years with results weighted for the NHANES sampling design (N=612).

	**FSH (mIU/mL)**		**AMH (ng/mL)**		**Estradiol (pg/mL)**		**Progesterone (ng/dL)**	
**PFAS (ng/mL)**	**% Change per IQR (95% CI)**	***p* Value**	**% Change per IQR (95% CI)**	***p* Value**	**% Change per IQR (95% CI)**	***p* Value**	**% Change per IQR (95% CI)**	***p* Value**
PFOS	31.7 (5.2, 64.9)	0.032	-12.9 (-30.4, 9.1)	0.236	-29.9 (-41.5, -16.1)	0.002	-47.4 (-63.3, -24.6)	0.003
n-PFOS	27.5 (4.1, 56.1)	0.033	-12.1 (-29.1, 9.1)	0.258	-27.6 (-39.5, -13.4)	0.003	-44.0 (-58.6, -24.1)	0.001
sm-PFOS	38.3 (3.6, 84.6)	0.044	-15.3 (-33.3, 7.5)	0.192	-29.3 (-42.3, -13.4)	0.004	-49.4 (-67.6, -20.8)	0.009

Model adjusted for age, ethnicity, family poverty income ratio, BMI, smoking status, drinking status, physical activity. Abbreviations: AMH: anti-Müllerian hormone; FSH: follicle-stimulating hormone; PFOS: perfluorooctane sulfonic acid.

Supplemental Table S4. Percent change in ln-serum sex hormone levels per IQR increase in PFAS Exposure Index concentrations (95% CI) among women aged ≥18 years in different models, with results weighted for the NHANES sampling design.

		**FSH (mIU/mL)**		**AMH (ng/mL)**		**Estradiol (pg/mL)**		**Progesterone (ng/dL)**	
**PFAS Exposure Index**	**N**	**% Change per IQR (95% CI)**	***p* Value**	**% Change per IQR (95% CI)**	***p* Value**	**% Change per IQR (95% CI)**	***p* Value**	**% Change per IQR (95% CI)**	***p* Value**
Primary Model	612	39.5 (8.9, 78.7)	0.019	-22.3 (-36.6, -4.9)	0.027	-34.6 (-47.8, -18.1)	0.002	-49.2 (-68.3, -18.5)	0.014
Model 2	610	24.8 (-2.3, 59.5)	0.097	-18.2 (-32.1, -1.4)	0.054	-28.7 (-43.7, -9.6)	0.013	-42.0 (-63.9, -7.0)	0.039
Model 3	609	39.2 (8.5, 78.1)	0.020	-21.2 (-35.6, -3.5)	0.037	-34.6 (-48.0, -17.7)	0.003	-49.4 (-69.1, -17.2)	0.016
Model 4	609	24.7 (-2.2, 59.0)	0.096	-17.2 (-31.4, 0.1)	0.070	-29.1 (-44.2, -10.1)	0.012	-42.8 (-64.9, -6.7)	0.041

Primary model: adjusted for age, ethnicity, family poverty income ratio, BMI, smoking status, drinking status, physical activity.

Sensitivity models additionally adjusted for serum albumin (Model 2), eGFR (Model 3), or both albumin and eGFR (Model 4).

Abbreviations: AMH: anti-Müllerian hormone; FSH: follicle-stimulating hormone.

Supplemental Table S5. Percent change in ln-serum sex hormone levels per IQR increase in the PFAS Exposure Index and principal component 1 (95% CI) among women aged ≥18 years across alternative models, with results weighted for the NHANES sampling design.

	**FSH (mIU/mL)**		**AMH (ng/mL)**		**Estradiol (pg/mL)**		**Progesterone (ng/dL)**	
	**% Change per IQR (95% CI)**	***p* Value**	**% Change per IQR (95% CI)**	***p* Value**	**% Change per IQR (95% CI)**	***p* Value**	**% Change per IQR (95% CI)**	***p* Value**
**PFAS Exposure Index**	39.5 (8.9, 78.7)	0.019	-22.3 (-36.6, -4.9)	0.027	-34.6 (-47.8, -18.1)	0.002	-49.2 (-68.3, -18.5)	0.014
**Principal component 1**	28.4 (10.2, 49.6)	0.019	-18.82 (-31.0, -5.5)	0.027	-31.9 (-44.3, -17.6)	0.002	-47.5 (-66.8, -15.9)	0.014

Primary model: adjusted for age, ethnicity, family poverty income ratio, BMI, smoking status, drinking status, physical activity.

Sensitivity models additionally adjusted for serum albumin (Model 2), eGFR (Model 3), or both albumin and eGFR (Model 4).

Abbreviations: AMH: anti-Müllerian hormone; FSH: follicle-stimulating hormone.

Supplemental Figure S1. Exploratory SEM illustrating the associations among the PFAS exposure index (exogenous variable), FSH (mIU/mL), estradiol (pg/mL), progesterone (ng/mL), and AMH (ng/mL). Paths represent hypothesized association structures after covariate adjustment. Panels display models including: (A) estradiol; (B) progesterone; (C) AMH; (D) estradiol and progesterone.

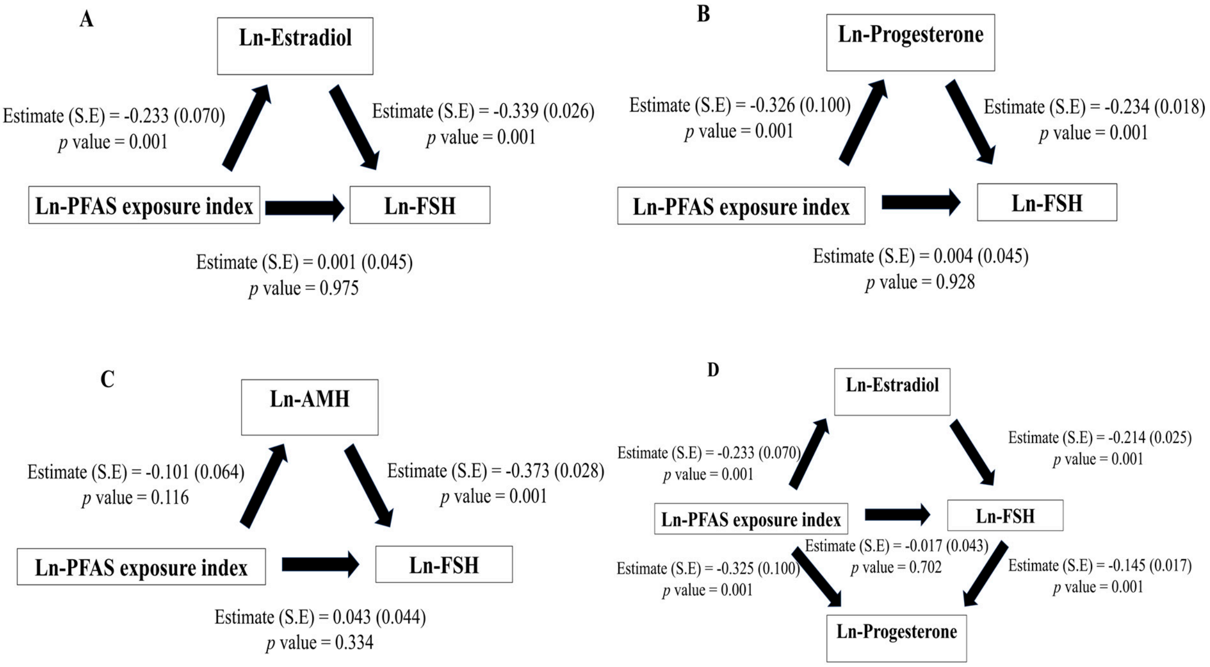

Supplement: Supplementary file 1 [file life-15-01923-s001.zip › life-3985171-supplementary.pdf]
